# Inhibition of fucosylation by 2-fluorofucose suppresses human liver cancer HepG2 cell proliferation and migration as well as tumor formation

**DOI:** 10.1038/s41598-017-11911-9

**Published:** 2017-09-14

**Authors:** Ying Zhou, Tomohiko Fukuda, Qinglei Hang, Sicong Hou, Tomoya Isaji, Akihiko Kameyama, Jianguo Gu

**Affiliations:** 1 Division of Regulatory Glycobiology, Institute of Molecular Biomembrane and Glycobiology, Tohoku Medical and Pharmaceutical University, 4-4-1 Komatsushima, Aoba-ku, Sendai, Miyagi 981-8558 Japan; 2 Department of Life Science and Biotechnology, National Institute of Advanced Industrial Science and Technology (AIST), 1-1-1 Umezono, Tsukuba, Ibaraki, 305-8568 Japan

## Abstract

Core fucosylation is one of the most important glycosylation events in the progression of liver cancer. For this study, we used an easily handled L-fucose analog, 2-fluoro-L-fucose (2FF), which interferes with the normal synthesis of GDP-fucose, and verified its potential roles in regulating core fucosylation and cell behavior in the HepG2 liver cancer cell line. Results obtained from lectin blot and flow cytometry analysis clearly showed that 2FF treatment dramatically inhibited core fucosylation, which was also confirmed via mass spectrometry analysis. Cell proliferation and integrin-mediated cell migration were significantly suppressed in cells treated with 2FF. We further analyzed cell colony formation in soft agar and tumor xenograft efficacy, and found that both were greatly suppressed in the 2FF-treated cells, compared with the control cells. Moreover, the treatment with 2FF decreased the core fucosylation levels of membrane glycoproteins such as EGF receptor and integrin β1, which in turn suppressed downstream signals that included phospho-EGFR, -AKT, -ERK, and -FAK. These results clearly described the roles of 2FF and the importance of core fucosylation in liver cancer progression, suggesting 2FF shows promise for use in the treatment of hepatoma.

## Introduction

Glycosylation is by far the most prolific form of protein modification in mammalian cells. Accumulating data have made it clear that glycan structures expressed on glycoproteins have essential roles in various biological processes such as inflammation, growth, differentiation, carcinogenesis, and cancer metastasis^1, 2^. Alteration during glycosylation is now regarded as a feature event in the progression of cancer. Among all forms of carbohydrate modification involved with the progression of cancer, fucosylation is considered one of the most important^3, 4^.

In regards to liver cancer, core fucosylation is a pre-eminent factor. Core fucosylation, also known as α1,6-fucosylation, is catalyzed by α1,6-fucosyltransferase (Fut8) to transfer fucose residue from guanosine 5/-diphosphate (GDP)-fucose to the innermost asparagine-linked GlcNAc via an α1,6 link, which is a process that has been implicated in the progression of liver cancer^5^. Early work by Breborowicz, J. *et al*. suggested that core fucosylated alpha-fetoprotein (AFP) was a reliable marker that could be used to distinguish hepatocellular carcinoma (HCC) from chronic hepatitis and liver cirrhosis^6^.

Recent studies have shown that core fucosylation is up-regulated both in the serum and on the cell surface of liver cancer cells^7, 8^, although contradictory results were also reported whereby an increased level of tetra-antennary N-glycan, rather than core fucosylation, was associated with hepatocellular carcinoma^9^. Consistently, Yamashita, F. *et al*. associated a high expression of core fucosylation with a poor prognosis^10^. Furthermore, our previous studies indicated that loss of Fut8 gene significantly suppressed liver regeneration^11^ and chemical-induced HCC^12^ via the down-regulation of several cell-signaling pathways. Taken together, these studies clearly show that core fucosylation plays crucial roles in liver cancer, which enabled us to predict that the inhibition of core fucosylation could provide a novel strategy for liver cancer therapy^9^.

How to inhibit core fucosylation in HCC? As described above, biosynthesis of core fucosylated *N*-glycans requires donor substrate GDP-fucose, *N*-glycans and Fut8. Fut8 is localized in the inner lumen of Golgi bodies, thus, conventional wisdom holds that an inhibitor of Fut8 cannot be efficiently delivered to a Golgi apparatus. On the other hand, GDP-fucose in the cytoplasm is known to be synthesized by two distinct pathways^13, 14^. One is called the *de novo* pathway, and this pathway converts GDP-mannose into GDP-fucose *via* enzymatic reactions catalyzed by GDP-mannose 4,6-dehydratase (GMD) and GDP-keto-6-deoxymannose 3, and 5-epimerase 4-reductase (FX)^15–17^. Blocking this pathway forces cells to make use of another pathway, which is called the salvage pathway. This pathway uses fucose kinases to convert L-fucose into GDP-fucose^18, 19^. GDP-fucose is then delivered into the Golgi apparatus via GDP-fucose transporters. Finally, GDP-fucose serves as a donor substrate and is transferred into the oligosaccharides of protein to synthesize core fucose by the action of Fut8^20^. Thus, the inhibition of GDP-fucose production is desirable in order to block fucosylation. Previous efforts to delete core fucosylation have focused mostly on the manipulation of Fut8 by knockout or knockdown of its gene. Additionally, there have been efforts to knockout the key enzymes for GDP-fucose production such as GMD and FX and impair the Golgi GDP-fucose transporter^21–25^. The methods described above, however, are not suitable for pharmacological application.

A variety of glycosyltransferases inhibitors have been developed, and mainly based on donor or accept substrates mimics^26^. Several GDP-fucose analogs have been reported to be inhibitors of FUTs^27, 28^. However, those charged groups (GDP portion) prevent uptake into cells, which limits their use in biological systems. On the other hand, a specific fluorinated analog of fucose, 2-fluoro-L-fucose (2FF), has been reported to easily enter cells via passive diffusion wherein it is metabolized into a corresponding donor substrate analog of GDP-fucose, GDP-2FF, via the salvage pathway^29–31^. Since GDP-2FF accumulates in cells, it will also lead to a shutdown of the *de* novo pathway that synthesizes natural GDP-fucose^29^. In fact, the addition of 2FF has efficiently suppressed the endogenous production of GDP-fucose, which dramatically inhibited the formation of fucosylation in both cancer and plant cells^31–33^. Therefore, 2FF has been used to reduce cell-surface fucosylated glycans such as Lewis antigens for E-selectin binding in colon carcinoma cells^31^, and has blocked core fucosylation in HL-60 cells^29^. However, the effect that 2FF exerts on liver cancer cells remains unclear.

In this study, we examined the effects of 2FF in live cancer HepG2 cells and further clarified the underlying molecular mechanisms. We found that treatment with 2FF greatly decreased core fucosylation levels and both suppressed downstream signaling and tumor formation, which suggested that 2FF might be a novel candidate for liver cancer therapy.

## Results

### 2FF suppressed fucosylation in HepG2 cells

**Figure 1:**
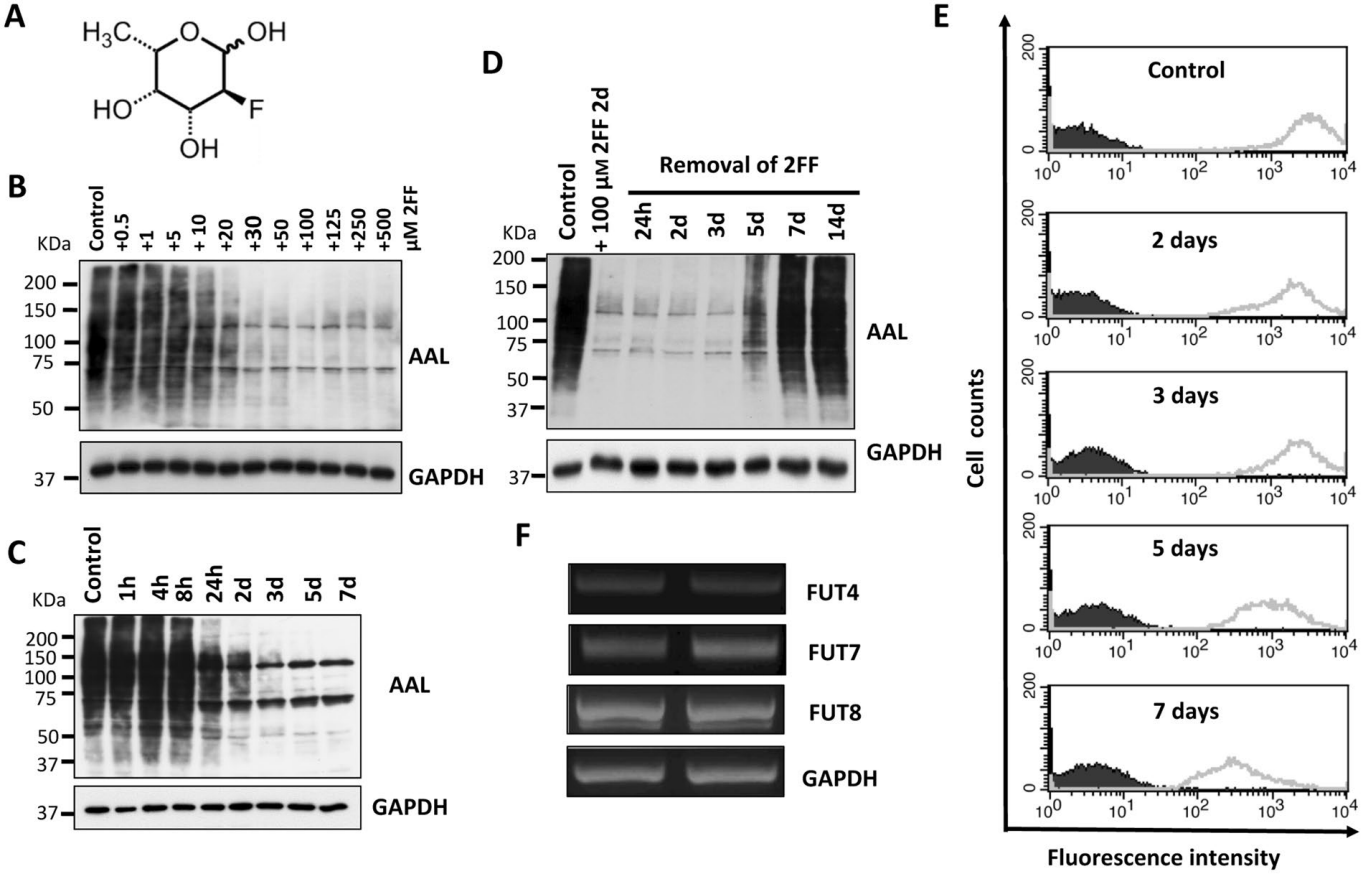
Treatment with 2FF suppressed fucosylation on glycoproteins. (**A**) The structure of 2FF as previously described^29^. (**B**) HepG2 cells were cultured with 2FF for 3 days at different concentrations, as indicated. Cell lysates were prepared, and then subjected to lectin blot analysis using AAL, which preferentially recognizes core fucose, as described in “Methods”. GAPDH was used as a loading control. Control refers to cells treated with the same amount of DMSO. (**C**) HepG2 cells were cultured in the presence of 100 μM 2FF for different time points (0 h-7 days). Equal amounts of proteins were probed with AAL lectin as described above. (**D**) HepG2 cells were cultured in the presence of 100 μM 2FF for 2 days, then the culture medium containing 2FF was removed and change into normal culture medium for different time points (24 h, 2d, 3d, 5d, 7d). (**E**) HepG2 cells were incubated with 100 μM 2FF at different time points, and then stained with (grey line) or without (dark line) biotin-conjugated AAL followed by streptavidin-conjugate Alexa Fluor 647 and subjected to flow cytometry analysis. (**F**) RT-PCR was carried out using total RNA extracted from HepG2 cells treated with or without 2FF to detect the expression level of representative genes involved in fucosylation. The expression level of GAPDH was examined as a loading control. The relative mRNA levels were normalized with the GAPDH control gene.

Several analogues of L-fucose have shown inhibitory effects on fucosylation. One such analogue is 2FF, as shown in Fig. 1A. To investigate whether 2FF also inhibits fucosylation levels in HepG2 hepatoma cells, we first carried out lectin blot testing to detect fucosylation levels by probing with *aleuria aurantia lectin* (AAL), a specific lectin that preferentially binds to Fuc α1-6GlcNAc^34^. The fucosylation levels were inhibited by 2FF in a dose-dependent manner. The inhibitory effects of 2FF were observed even at a final concentration at 10 μM, and remarkably appeared at 100 μM (Fig. 1B). In addition, time course studies have indicated that the effect of 2FF persisted for more than 7 days without further adding 2FF (Fig. 1C). After the removal of 2FF, its effect at least lasted for another 3 days (Fig. 1D). These alterations in the fucosylation expression on the cell surface were further confirmed by flow cytometric analysis using AAL lectin. The reactive abilities of AAL lectin were persistently decreased in the HepG2 cells during 7-day culture in the presence of 2FF, compared with the control cells cultured without 2FF (Fig. 1E). To explore whether the decreased fucosylation was due to a suppression of fucosyltrasferase gene expression by 2FF, we performed semi-quantitative RT-PCR to detect typical fucosylation related genes such as α1,3-fucosyltransferase 4 (Fut4), α1,3-fucosyltransferase 7 (Fut7), and Fut8 because 2FF was known to inhibit not only the products of Fut8 but also the products of Fut4 and Fut7 in HL-60 cells^29^. As shown in Fig. 1F, the treatment with 2FF did not significantly affect gene expression level. These data again indicated that 2FF strongly down-regulates fucosylation levels via an interruption of intracellular GDP-fucose synthesis, and/or that GDP-2FF may inhibit fucosyltransferases, which includes Fut8, in HepG2 cells.

### Alteration of the N-linked glycans of HepG2 cells treated with 2FF

**Figure 2:**
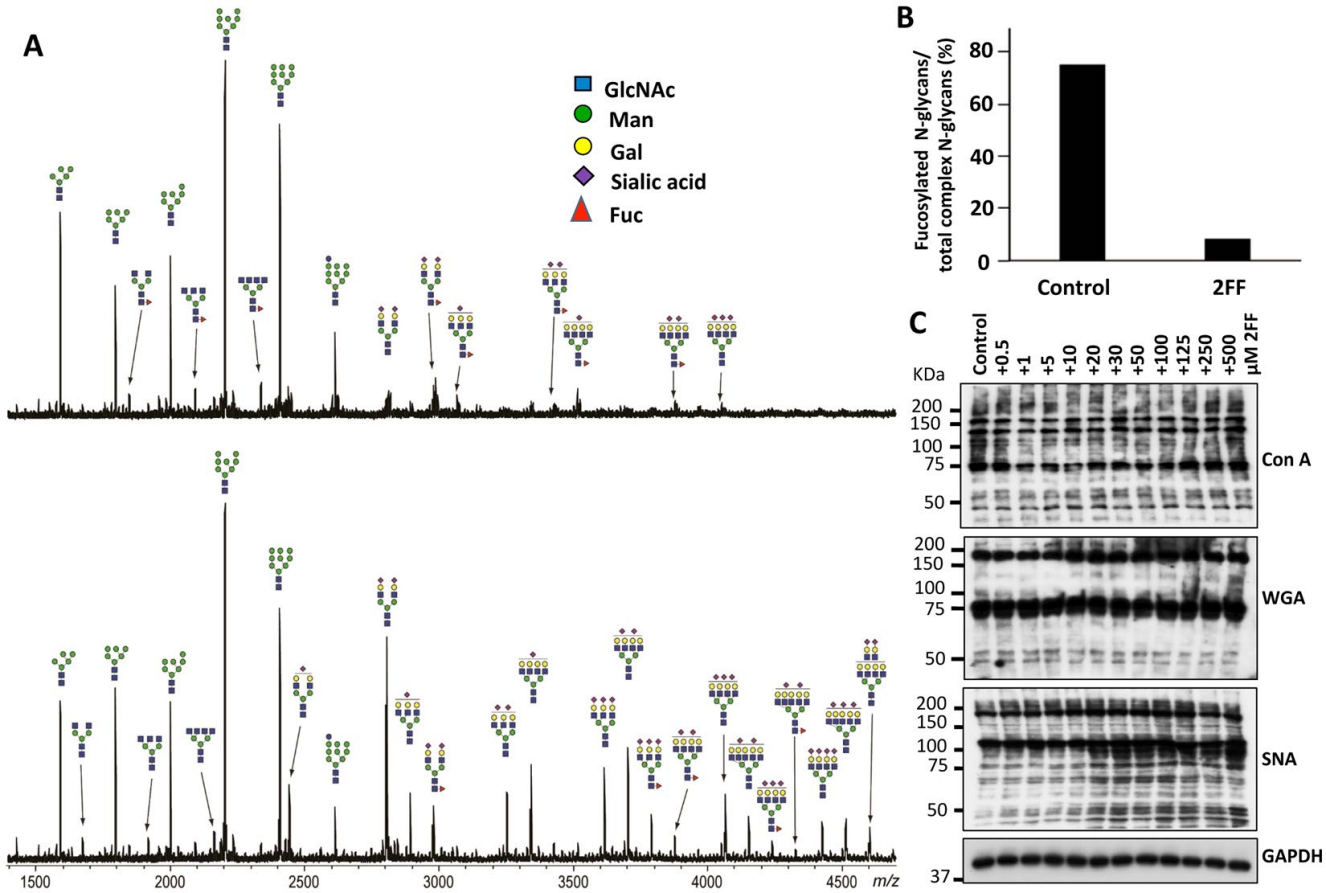
Comparison of N-glycan profiles in HepG2 cells treated with and without 2FF. (**A**) MS spectrum of the permethylated glycans from the cells treated with DMSO only (upper) and DMSO containing 2FF (lower). (**B**) Percentages of fucosylated complex glycans in total complex glycans from HepG2 cells. The values were calculated from peak areas in the range of m/z 1,500-3,500 of the MS spectra. (**C**) HepG2 cells were cultured with 2FF for 3 days at different concentrations as indicated, and then equal amounts of cell lysates were probed with Con A, which specifically recognizes alpha-linked mannose and glucose; WGA, which selectively binds to N-Acetyl glucosamine (GlcNAc) and SNA, which specifically recognizes α 2, 6 sialylation. GAPDH was used as a loading control.

To further verify the effects of 2FF in regulating carbohydrate structures, the *N*-glycan profiles of those cells were analyzed by mass spectrometry. MALDI-TOF mass spectrometry of *N*-glycans clearly supported the suppression of core fucosylation by treating HepG2 cells with 2FF (Fig. 2A). Without 2FF, fucosylated complex N-glycans were accompanied by large signals from high mannose-type glycans (Fig. 2A upper). These fucosylated glycans, mostly core fucosylated glycans, were considerably displaced by corresponding afucosylated glycans in 2FF-treated HepG2 cells (Fig. 2A lower). The percentages of fucosylated complex glycans among all complex glycans in an m/z range of from 1,500 to 3,500 were reduced from 75 to 8% by treatment with 2FF (Fig. 2B). Curiously, sialylated complex glycans were observed in an m/z of more than 3,500 in both measurements of the MS spectra. Afucosylated sialylated glycans were also significantly increased by treatment with 2FF. In fact, upon 2FF treatment, reactivity against both *concanavalin A* (ConA) and *wheat germ agglutinin* (WGA) showed no change. An SNA lectin blot showed a tendency whereby sialylation was up-regulated upon 2FF treatment in a dose-dependent manner (Fig. 2C). That phenomenon could be interpreted as sialyation is up-regulated by a lack of terminal fucosylation, which was blocked by 2FF, since fucosyltransferase and sialytransferase can compete for the same terminal structure as an accepted substrate^29^. These results clearly demonstrated that fucosylation, particularly core fucosylation, is down-regulated via 2FF treatment to HepG2 cells.

### Treatment with 2FF inhibited cell proliferation and colony formation

**Figure 3:**
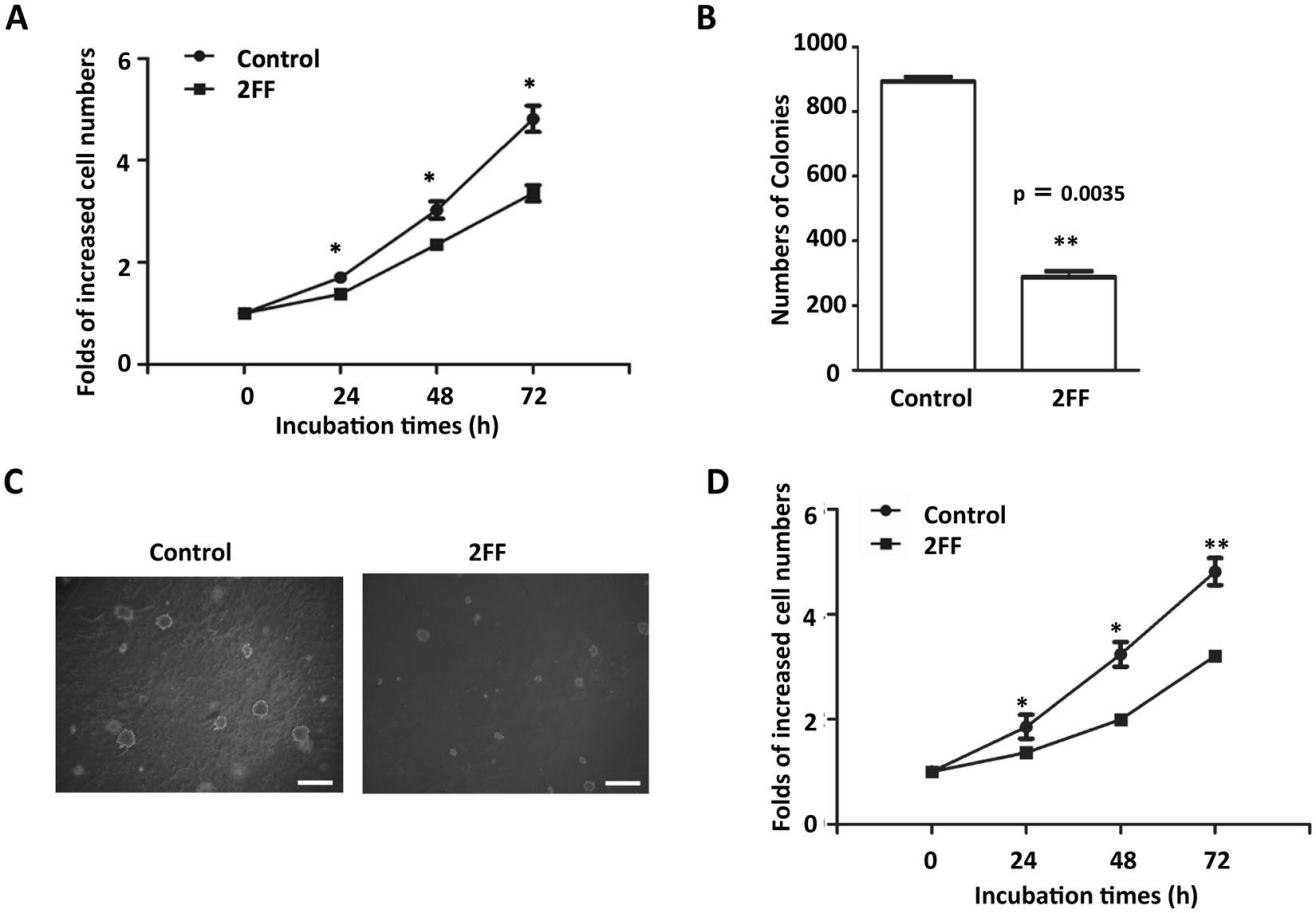
Effects of 2FF treatment on cell proliferation. HepG2 and Hela cells were pre-treated with or without 100 μM 2FF for 3 days and then starved for 24 h and replaced with DMEM containing 3% FBS to assay cell proliferation. (**A**) The pre-treated cells were incubated in 2FF containing medium for another 4 days. The time point at the replacement was deemed as 0 h. Cells at the same areas were photographed and counted every 24 h. The results are shown as fold changes relative to the numbers of those at 0 h, and presented as the mean ± s.e.m. (*p < 0.05 by t-test). (**B**) Soft agars were prepared as described in the “Methods”. Controls and 2FF-treated HepG2 cells (5,000 per/well) were cultured on the soft agar for 3 weeks. The colonies that formed were stained with crystal violet. The stained colonies were counted (**p < 0.01 by t-test). Three independent experiments were performed. **(C)** Representative images showed different sizes of colonies treated with or without 2FF. Scale bars indicate 500 μm. (**D**) Pre-treated Hela cells were incubated in 2FF containing medium for another 4 days. The time point at the replacement was deemed as 0 h. Cells were photographed at the same areas and counted every 24 h. The results are shown as fold changes relative to the numbers of those at 0 h, and presented as the mean ± s.e.m. (*p < 0.05 by t-test, **p < 0.01 by t-test).

In consideration of the importance of core fucosylation in physiological and pathological functions^11, 12, 35^, we speculated a potential role for 2FF in the regulation of cell proliferation. To test this hypothesis, we investigated the proliferation of HepG2 cells, which were pre-treated with or without 2FF for 3 days followed by 24 h serum-starvation. As shown in Fig. 3A, cell proliferation was significantly suppressed following treatment with 2FF, compared with control cells without 2FF treatment (Fig. 3A). There was no significant difference in cell viabilities between the control and 2FF-treated cells (data not shown). This phenomenon was also observed in Hela cells (Fig. 3D). The ability to grow in an anchorage-independent manner has always been regarded as one of the hallmarks of cancer, and this ability is believed closely related to cell proliferation and cell-survival signals. Therefore, we performed a clonogenicity study using a soft agar assay. Colony formation abilities were significantly suppressed in the HepG2 cells treated with 2FF, compared with control cells (Fig. 3B). Each colony size treated with 2FF was much smaller than the control without 2FF (Fig. 3C). In contrast, Rillahan, C.D. *et al*., reported that inhibition of fucosylation in HL-60, Ramos, or CHO-K1 cells did not significantly affect cell proliferation^29^. These contradictory data could be speculated that the different phenotypes could be due to cell types. Since different cell types differentially express growth factor receptors to control cell proliferation, for example, HepG2 cells highly express EGFR and c-Met, while HL-60 cells and CHO-K1 cells do not express EGFR^36, 37^. In fact, HL-60 cells abundantly express insulin-like growth factor I receptor (IGF-IR), which may be modified by different glycans compared with those on EGFR, to contribute to the cell growth^38, 39^. Furthermore, even same receptor may also be modified by different glycans in different cells.

### 2FF suppressed integrin-mediated cell migration

**Figure 4:**
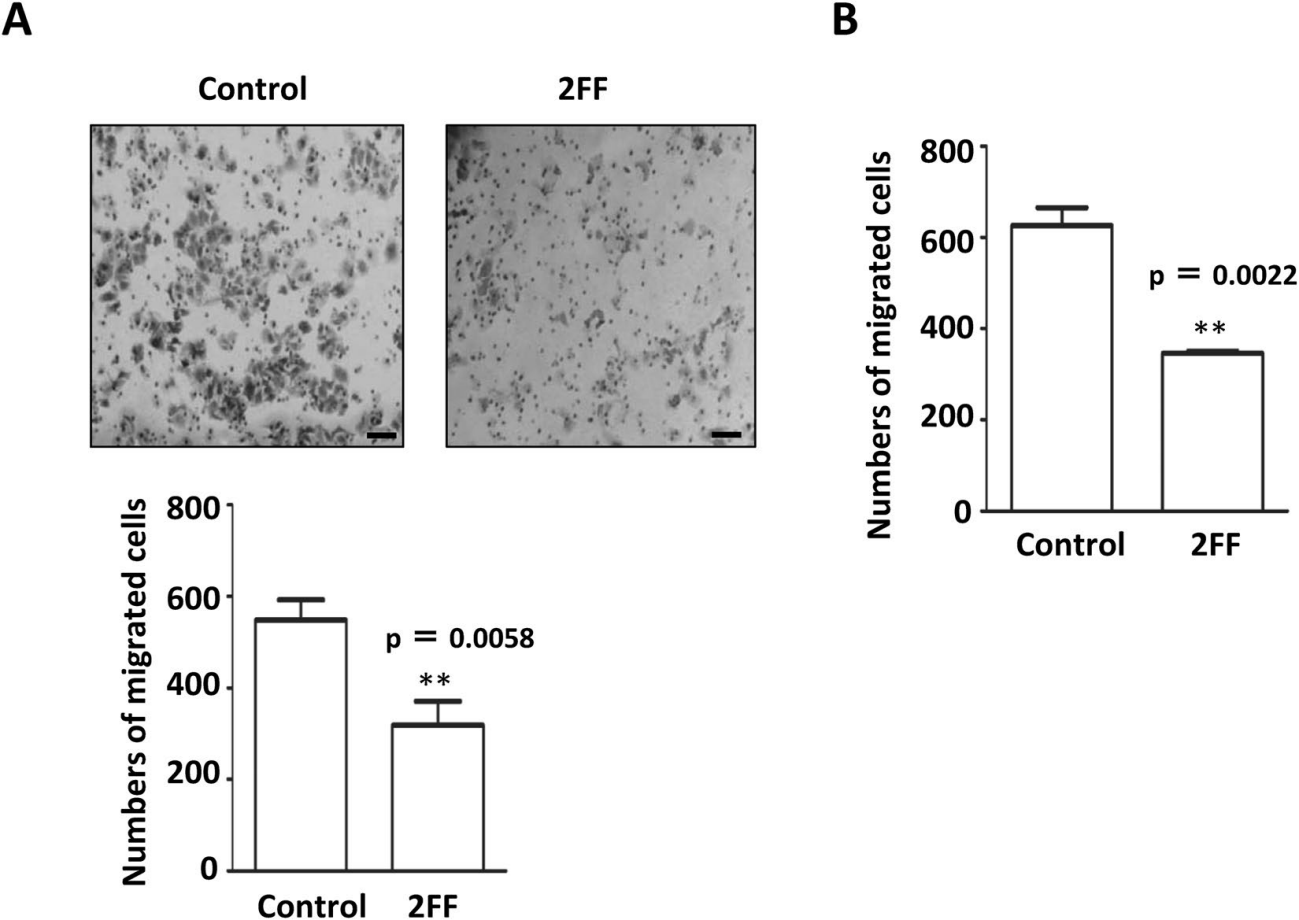
Effects of 2FF treatment on integrin-mediated cell migration. (**A**) The migration abilities of HepG2 cells toward FN were detected by transwell assay as described in “Methods”. HepG2 cells pre-treated with and without 2FF for 3 days were seeded into the top chambers of a transwell. After incubation for 24 h, the migrated cells were fixed and stained with 0.5% crystal violet. Representative images of the bottom surface were captured using phase-contrast microscopy (upper). The numbers of migrated cells in nine random microscopic fields (×200) were counted and averaged. Data represent the mean ± s.e.m. of three independent experiments (lower) (**p < 0.01 by t-test). Scale bars indicate 50 μm. (**B**) The migration abilities of Hela cells toward FN were detected. Hela cells pre-treated with or without 2FF for 3 days were seeded into the top chambers of the transwell, and incubated for 4 h. The migrated cells were calculated as described above. Data represented the mean ± s.e.m. of three independent experiments (**p < 0.01 by t-test).

As described above, 2FF treatment had a major impact on cell growth. Then, we used a transwell assay to examine the migratory ability of HepG2 cells following 2FF treatment. As shown in Fig. 4A, the integrin-mediated cell migration was significantly inhibited in 2FF-treated cells compared with control cells. In a similar manner, the migratory ability that was suppressed by 2FF was also observed in Hela cells (Fig. 4B). Collectively, these data strongly suggested that 2FF could effectively inhibit cell proliferation and cell migration *in vitro*.

### 2FF inhibited core fucosylation of EGFR and integrin β1 and the related intracellular signaling

*N*-Glycosylation is known to be essential for the functional expression of membrane glycoproteins such as EGFR and integrins, and regulates several functions: cell-surface expression, dimerization, endocytosis and specific ligand binding^40, 41^. Recently, we showed that a loss of core fucosylation via knockout of the Fut8 gene decreased the interaction between EGFR and its ligand, including downstream signaling, which in turn suppressed cell proliferation^11, 12^. In addition, the deletion of core fucosylation significantly suppressed integrin α3β1-mediated cell migration^42^. Therefore, issues having to do with the down-regulation of cell proliferation and migration were related in these molecules.

**Figure 5:**
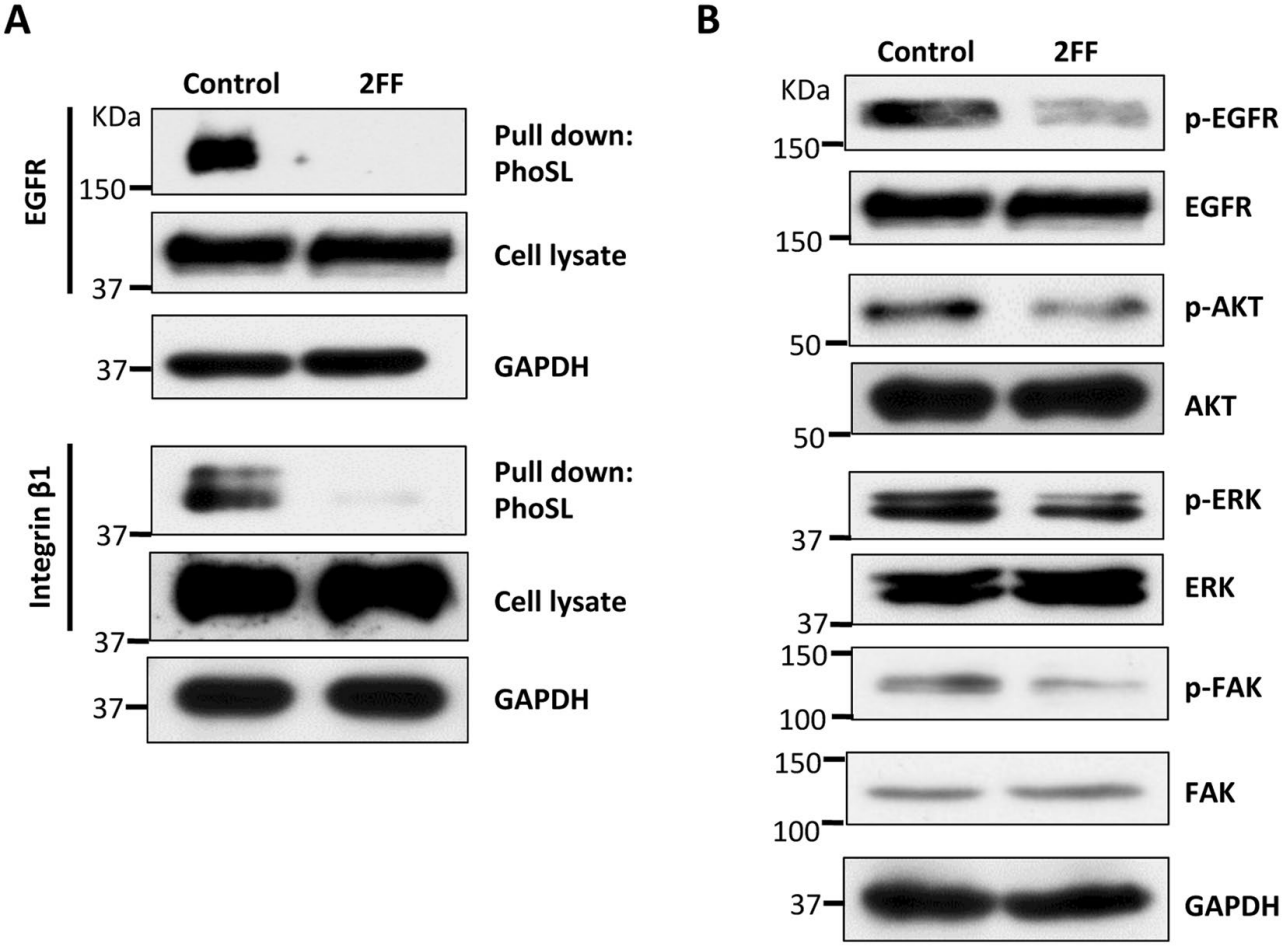
Treatment with 2FF suppressed core fucosylation on EGFR and integrin β1, as well as down stream signaling. HepG2 cells were cultured with and without (control) 2FF for 3 days, and harvested for lectin pull down assay or immunoblotting as described in “Methods”. (**A**) The cell lysates were pooled and incubated with 10 μl of indicated bead-bound PhoSL, which specifically recognizes core fucose. The lectin precipitated samples and equal amounts of cell lysates were then subjected to SDS-PAGE, and probed with anti-EGFR (upper panel) and anti-integrin β1 (lower panel) antibodies. GAPDH were used as a loading control. (**B**) Cell lysates were immunoblotted with anti-phospho-EGFR, anti-EGFR, anti-phospho-AKT, anti-AKT, anti-phospho-ERK1/2, anti-ERK1/2, anti-phospho-FAK, and anti-FAK antibodies, respectively. GAPDH was used as a loading control.

First, we performed a pull down assay using the biotinylated PhoSL, which preferentially recognizes core fucosylated N-glycans, to examine the effect of 2FF on the core fucosylation of EGFR and integrin β1^43^. As shown in Fig. 5A, core fucosylation was expressed on EGFR (upper panel) and integrin β1 (lower panel) in the control cells, but not in the 2FF-treated HepG2 cells, suggesting that 2FF efficiently blocked core fucosylation in these membrane proteins. Then, we asked whether 2FF affected intracellular signaling. To address this point, we examined the expression levels of phospho-EGFR and its downstream signaling such as phospho-AKT and phospho-ERK, as well as integrin’s downstream signal phospho-FAK. Interestingly, all of these signals were suppressed in the 2FF-treated HepG2 cells, compared with the control cells (Fig. 5B). Based on these observations, it is not difficult to conclude that the decreased core fucosylation by 2FF might impair the biological functions of some important membrane receptors such as EGFR and integrin β1, which subsequently inhibits intracellular signaling.

### Inhibitory effects of 2FF on tumorigenesis and core fucosylation *in vivo*

**Figure 6:**
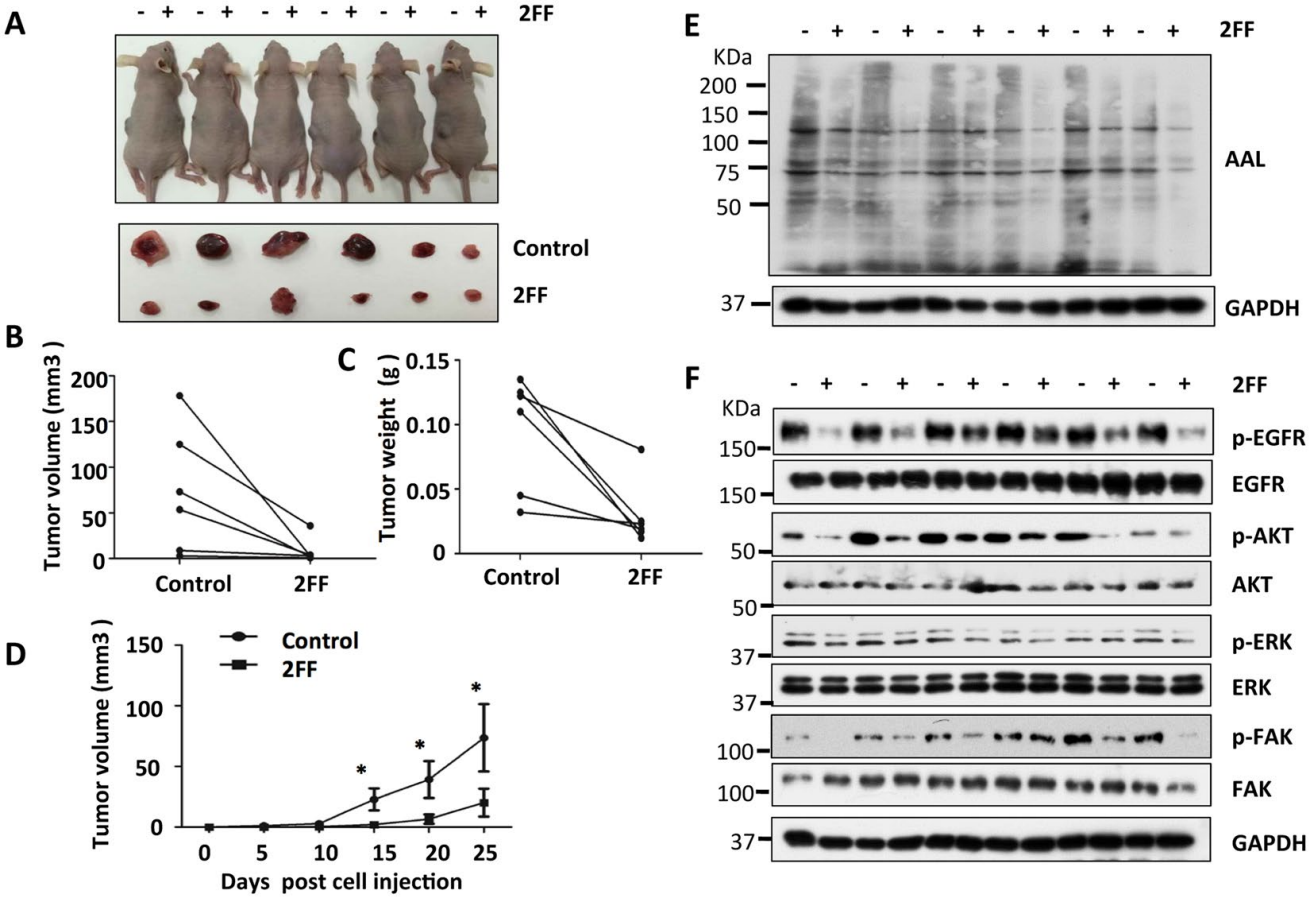
Effects of 2FF on tumor growth and fucosylation *in vivo*. HepG2 cells (4 × 10^6^) were inoculated into both the left (control cells, -) and right posterior flanks (2FF-treated cells, + ) of nude BALB/c-nu mice as described in “Methods”. (**A**) Photographs illustrate the representative features of tumor growth 25 days after injection (upper panel), and each corresponding pair of dissected tumor tissues (lower panel). Changes in each of the corresponding pair of tumor volumes (**B**) and weights (**C**) were also measured. (**D**) Tumor growth curves were measured every 5 days (n = 6, *p < 0.05 by t-test). (**E**) Tumor tissues were homogenized and lysates were blotted with AAL lectin. GAPDH was used as a loading control. (**F**) The lysates from tumor tissues were immunoblotted with anti-phospho-EGFR, anti-EGFR, anti-phospho-AKT, anti-AKT, anti-phospho-ERK1/2, anti-ERK1/2, anti-phospho-FAK, and anti-FAK antibodies, respectively. GAPDH was used as a loading control.

Based on the inhibitory effects of 2FF on malignant phenotypes observed *in vitro*, we used a well-established xenograft tumor model to further examine the tumorigenicity of HepG2 cells *in vivo*. The same amounts of control and 2FF pre-treated HepG2 cells (4 × 10^6^) were injected subcutaneously into the left and right flanks of each mouse, respectively. Based on the inhibitory effect of 2FF on fucosylation that lasted for 7 days at least (Fig. 1C); 100 μl of the DMEM solution containing with 2FF at 100 μM were directly injected into each tumor tissue from three directions after the inoculation for 7 days, and once a week for 3 weeks. All the mice were euthanized at 25 days after the first injection of 2FF. Tumor tissues were isolated, photographed and then weighed. Interestingly, significant decreases were noted in both the size (Fig. 6A,B) and weight (Fig. 6C) of tumor tissues in the 2FF-treated side, compared with the control side 25 days after inoculation. The time course of tumor growth also clearly showed the inhibitory effects of 2FF on tumor formation (Fig. 6D). Furthermore, AAL lectin blot showed that the levels of core fucosylation were apparently decreased in the tumor tissues that grew from the HepG2 cells treated with 2FF, compared with the controls (Fig. 6E). Importantly, in accordance with the results *in vitro* (Fig. 5), the cellular signals including p-EGFR, p-AKT, p-ERK and p-FAK in the 2FF-treated tumor tissues were decreased compared with those in the controls treated without 2FF (Fig. 6F). These results addressed the efficient effects of 2FF *in vivo*.

## Discussion

Liver cancer ranks as the second leading cause of death from cancer^44^. Effective therapy for liver cancer remains one of the biggest challenges for public health^45^. Increased levels of core fucosylation, usually higher expressions of either Fut8 or GDP-fucose, have lately been recognized as reliable indicators for liver cancer^3^. Therefore, regulation of the core fucosylation of *N*-glycans can serve as an essential strategy for liver cancer therapy. For example, knockout of the Fut8 gene in mice strongly suppressed a chemical-induced hepatocellular carcinoma^12^. Also, down regulation of the Fut8 expression by miRNAs inhibited the progression of liver cancer^35^. In addition, an abrogation of ability to form or transport GDP-fucose was also worked^9^. Those findings prompted us to investigate whether there is a pharmacological approach that could block core fucosylation in liver cancer cells.

In this study, we used the easily handled 2FF, an analog of fucose, to inhibit GDP-fucose formation. Considered a universal inhibitor of fucosylation, 2FF is used to reduce cell-surface fucosylated glycans such as Lewis antigens and core fucosylated *N*-glycans^29^. Our results, as determined by western blot and flow cytometry in liver cancer cells, also confirmed this conclusion (Fig. 1B–E). The inhibitory effects on fucosylated glycans did not seem to be due to an inhibition of the expression levels of fucosyltransferases, since there were no significant changes between cells treated with or without 2FF in the mRNA levels of typical fucosylation-related genes such as Fut4, Fut7 and Fut8, which produce Lewis X (Le^X^) or sialyl Lewis X (SLe^X^) and core fucosylated *N*-glycans, respectively (Fig. 1F). Surprisingly, changes in the *N*-glycan structures analyzed by mass spectrometry have shown that the majority of fucosylation in HepG2 cells is core fucosylation, while α1,2; α1,3 or α1,4 fucosylated *N*-glycans were at undetectable levels (Fig. 2). However, unexpectedly, sialylation levels seemed to be enhanced in the 2FF-treated cells (Fig. 2). It could be partly explained by that fucosyltransferases and sialyltransferases may compete for the same terminal glycan structures as an accepted substrate^29^. In fact, Rillahan, *et al*. reported that a treatment with the sialylation inhibitor resulted in a complete loss of sialic acids and a notable increase in overall fucosylation. Conversely, knockout of Fut9, a key enzyme for the synthesis of Le^X^ or SLe^X^, resulted in an increase in sialylation^46^. The detailed molecular mechanisms are required for further study. Thus, it is not difficult to speculate that the effects of 2FF in HepG2 cells share similar phenomena as observed in Fut8 deficient liver cells. As expected, the treatment with 2FF significantly suppressed cell proliferation and colony formation (Fig. 3)^11, 12^. Alterations of glycans can have a significant impact on cell proliferation, migration and invasion, all of which contribute to the transformation progress and subsequent metastasis of malignancy^47–49^. In order to further understand the link between 2FF-induced core fucosylation deficiency and cellular progression, we examined cell migration, and found that 2FF inhibits integrin-mediated cell migration (Fig. 4). These results clearly indicated that 2FF effectively suppresses both cell proliferation and cell migration.

Previously, we reported that core fucosylation was required for the binding of EGF to a receptor and for the regulation of EGFR-mediated intracellular signaling in embryonic fibroblast cells^50^. In addition, the attenuated EGFR-mediated signaling was confirmed in Fut8 knockout HepG2 cells^12^. Conversely, the incorporation of core fucose by Fut8 promoted EGFR dimerization and phosphorylation in lung cancer cells^51^. Furthermore, *N*-glycosylation is now known as a critical determinant of EGFR conformation, and specifically the orientation of the EGFR ectodomain relative to the membrane^52^. In agreement with these observations, this study showed that treatment with 2FF also decreased the levels of phospho-EGFR and downstream signaling such as phospho-ERK and phospho-AKT (Fig. 5B). It is reasonable to conclude that the inhibitory effect of 2FF on cell proliferation can at least be partially mediated via attenuated EGFR-mediated signaling. On the other hand, integrins are known to associate with EGFR in order to cooperatively regulate cell proliferation, migration, differentiation and survival in fibroblasts and epithelial cells^53^. Also, with EGFR they share some of the elements in signal pathways such as AKT and ERK^54^. Among the various types of integrins, integrin β1 plays crucial roles in cell migration and adhesion^55–57^. A knockout of β1 has sufficiently inhibited cell migration and affected the EFGR-mediated cell proliferation in a cell density-dependent manner in MDA-MB231 breast cancer cells^58^. In the present study, 2FF suppressed cell migration and the attenuated integrin-mediated specific downstream signaling of phospho-FAK (Fig. 5B), which is responsible for integrin-mediated cell migration^59, 60^. In fact, we previously reported that deletion of Fut8 significantly suppressed integrin α3β1-mediated cell migration^42^. Nonetheless, we cannot exclude the possibility that 2FF may influence the functions of other glycoproteins, since core fucose widely exists on the surfaces of cells.

It was also worth noting that although we addressed the importance of core fucosylation in cell behaviors since 2FF mainly inhibited core fucosylation in HepG2 cells, we could not conclude that other fucosylations expressed at marginal levels were unimportant. Previous studies have shown that oral administration of 2FF inhibits neutrophil extravasation *via* the suppression of Lewis antigen expression and delays tumor growth in immune deficient mice^31^. In order to further address the effects of 2FF *in vivo*, we subcutaneously inoculated HepG2 cells pretreated with 2FF (intratumoral administration before treatment) into nude mice. After tumors were formed, 2FF solutions were directly injected into each tumor tissue. Consistently, treatment with 2FF prevented tumorigenesis as well as core fucosylation *in vivo* (Fig. 6). These data strongly suggested that the chemical compound 2FF is easy to handle and exerts a potential inhibitory effect on tumor growth.

In addition, we also cannot exclude the potential effects on sialylation. As described above, sialylation and fucosylation can be regulated mutually. However, the phenomenon, an increase in sialylation in the 2FF-treated cells, observed in the present study could not be simply explained by mutual regulation. The increased sialylation may associate with some unknown mechanisms, and could also influences cell biological functions, which requires further study. In fact, an aberrant expression of sialylation has long been associated with metastatic cell behaviors such as invasion and enhanced cell survival, and the increased α2,6 sialylation on *N*-glycans catalyzed by β-galactoside α2,6 sialyltranferase 1 (ST6Gal I) is frequently observed in many types of the cancers^61, 62^.

In summary, our study provided evidence that 2FF inhibited core fucosylation in the HepG2 cells of cell-surface glycoproteins, which in turn inhibited cellular signaling for cell proliferation and migration as well as for tumor growth. We believe that 2FF shows promise as a therapeutic compound for hepatoma and other cancers.

## Methods

### Antibodies and reagents

Experiments were performed using the following antibodies: Rabbit antibodies against EGFR, p-EGFR, AKT, p-AKT, ERK1/2, and p-ERK1/2 were purchased from Cell Signaling Technology (Danvers, MA, USA); Mouse antibodies against integrin β1, FAK and p-FAK were purchased from BD Biosciences (Franklin Lakes, NJ, USA); Rabbit antibody against glyceraldehyde-3-phosphate dehydrogenase (GAPDH) was purchased from Santa Cruze Biotechnology (Santa Cruz, CA, USA). Peroxidase-conjugated goat antibodies against rabbit and mouse IgG were obtained from Cell Signaling Technology and Promega, respectively. Biotinylated *aleuria aurantia lectin* (AAL), *concanavalin A* (ConA) and *wheat germ agglutinin* (WGA) were purchased from Seikagaku Crop (Tokyo, Japan). Biotinylated Pholiota Squarrosa lectin (PhoSL), which specifically recognizes core fucosylated N-glycans, was a generous gift from Dr. Yuka Kobayashi (J-oil Mills, Tokyo, Japan). Alexa Fluor® 647 goat anti-mouse secondary antibody and fetal bovine serum (FBS) were purchased from Invitrogen (Life Technologies). 2FF was purchased from Synchem, Inc., IL, USA. Fibronectin (FN) and dimethyl sulfoxide (DMSO) were obtained from sigma (St. Louis, MO, USA); and, Dulbecco’s modified Eagle’s medium (DMEM) was purchased from Gibco (Grand Island, NY, USA).

### Cell culture and treatment

Human hepatoma HepG2 and Hela cell lines were purchased from the RIKEN cell bank (Japan). Cells were cultured in DMEM containing 10% FBS and incubated at 37 °C in a humidified atmosphere with 5% CO_2_. The cells were incubated with and without 2FF dissolved in DMSO for indicated times. The final concentrations of 2FF were diluted with culture media at 0.5~500 μM. The same amounts of DMSO were used as controls.

### Western blot and lectin blot assay

Cells cultured in the presence or absence of 2FF under different conditions, as indicated, were harvested, washed by cold PBS and then scraped off culture plates with lysis buffer containing 20 mM Tris-HCl (pH = 7.4), 1% Triton X-100, 150 mM NaCl, and protease and phosphatase inhibitors (Nacalai Tesque). After incubation for 15 min at 4 °C, cell debris were removed by centrifugation for 10 min at 13,000 g. Protein concentrations were measured using a BCA protein assay kit (Pierce). Equal amounts for each sample were run on 6, 7.5 or 10% SDS-PAGE gels, as indicated.

For the pull-down assay, 500 μg of cell lysates were collected and incubated with 10 μL FG streptavidin beads (Tamagawa Seiki Co., Tokyo, Japan) and 2 μg biotinylated PhoSL for 2 h at 4 °C. The precipitates were then washed by cold lysis buffer and boiled in SDS loading buffer. Resolved proteins were then transferred onto a PVDF membrane (Millipore) for detection.

For Western blot assay, the membranes were blocked with 5% skim milk in TBS containing 0.1% Tween-20 (TBST) for 1 h at room temperature (RT) and then probed with specific antibodies at 4 °C overnight. Detection was achieved using secondary HRP-conjugated IgG against rabbit or mouse IgG and finally visualized using the ECL system (Amersham).

For lectin blot assay, the membranes were blocked with 3% bovine serum albumin (BSA) in TBST followed by specific lectins. The bands were finally visualized using a Vectastain ABC Kit (Vector Laboratories).

### RT-PCR

Total RNAs of HepG2 cells treated with and without 2FF were extracted by Trizol reagent (Invitrogen). 1 μg of total RNAs were reverse-transcribed using a Prime Script RT Reagent kit with gDNA Eraser (Takara, Japan). The specific primers used for the PCR amplification were as follows: Fut4, forward, 5′-ACTACCACCAACTGAGCCAACATGTGA-3′, reverse, 5′-AAGGAGGTGATGTGGACAGCGTAG-3′;

Fut7, forward, 5′-AGTACCGCTTCTACCTGTCCTTTGAGAA-3′, reverse, 5′-AGCCTGTCACGCCAGGCAAAGAA-3′; and, Fut8, forward, 5′-CACTTGGTACGAGATAATGAC-3′, reverse, 5′-CACATGATGGAGCTGACAGCC-3′. The GAPDH mRNA served as the control. The obtained reaction products were then placed into 2% agarose gels containing ethidium bromide for electrophoresis.

### Flow cytometry analysis

Cells were harvested and adjusted to a concentration of 1-5 × 10^6^ cells/mL in cold PBS. Then cells were stained with biotinylated AAL for 1 h on ice, followed by streptavidin-conjugate Alexa Fluor 647 for 1 h in the dark on ice. A negative control was prepared without AAL. After incubation, cells were washed 3 times with ice-cold PBS, and then analyzed using a FACSCalibur flow cytometer (BD Biosciences).

### Mass spectrometry

Membrane fractions of cells were prepared as described previously^63^. Pellets were dissolved in 100 μL of 0.1 M Tris-HCl (pH = 8.6), then 20 μL of 200 mM DTT in 1 M Tris-HCl (pH = 8.6) containing 1% SDS was added with heating to 80 °C for 10 min. After cooling to RT, 40 μL of 5% Nonidet P-40 and 15 μL of distilled water were added to the mixture. Glycopeptidase F (16 mU, Takara Bio Inc.), an amidohydrolase that removes intact *N*-glycans from glycoproteins, was added to the solution, which was then incubated at 37 °C for 16 h. After the addition of 234 μL of ethanol to the solution, the mixtures were cooled to −20 °C and kept in a freezer for 20 min followed by centrifugation at 15,000 rpm for 20 min. Supernatants were transferred into new microtubes and dried via a centrifuge evaporator. The residues were dissolved in 200 μL of water, and then loaded onto a solid-phase extraction cartridge (Sep-Pak C18, 50 mg, Waters Corp) and washed with 1 mL water. The flow-through and washings were combined and applied to a HyperSep Hypercarb SPE cartridge (25 mg). After washing with 1 mL water and then with 1 mL 0.1% TFA, glycans were eluted with 1 mL of 50% acetonitrile containing 0.1% TFA. The eluted solution was dried *in vacuo*, and then permethylated as described previously^63^. Mass spectrometry was performed using a MALDI-TOF mass spectrometer (Ultraflex). Ions were generated using a 337-nm nitrogen laser and were accelerated to 20 kV. All spectra were obtained with a delayed extraction of 140 ns in a reflectron mode, and were the result of a signal averaging of 300 laser shots. For sample preparation, 2, 5-dihydroxybenzoic acid was used as a matrix.

### Cell growth assay

HepG2 and Hela cells were pre-treated with or without 2FF at a final concentration of 100 μM for 3 days followed by 24 h serum-starvation. Then, 3 × 10^3^ cells were then seeded into 6-cm dishes and cultured under 3% FBS media with or without 100 μM 2FF. Cells were photographed in the same area when the indicated times were reached. Cell numbers at each time point were counted and normalized to those at 0 h when the cell culture media were replaced with 3% FBS.

### Soft agar assay

A total of 1 mL 0.5% base agar in complete culture medium was added into each 6-cm dish and set aside for 5 min to allow the agars to solidify. The pre-treated cells described above were then mixed with the 0.33% agarose (5,000 cells/mL) and layered onto the base agar. Agar dishes were incubated at 37 °C and fed with complete culture medium. Culture media were changed every 4–5 days. Three weeks later, agar dishes were stained with 0.005% crystal violet overnight. The colony numbers were counted from three independent plates. Representative photographs were taken.

### Transwell cell migration assays

Cell migration assays were performed using a transwell chamber (BD BioCoat^TM^ control inserts, 8.0-mm inserts; BD Biosciences) according to the manufacturer’s instructions. Briefly, the bottom sides of transwells were coated with 10 μg/mL fibronectin (FN) and let stand at 4 °C overnight. Cells were harvested by trypsin containing 1 mM EDTA and re-suspended in the serum-free DMEM medium at a concentration of 1 × 10^5^ cells/mL for HepG2 and 4 × 10^4^ cells/mL for Hela. Then, 500 μl of suspension was added to the upper chamber and cells were allowed to migrate to the lower chamber containing 500 μL complete medium at 37 °C. At the end of the cell migration, the filter side of the upper chamber was cleaned using cotton swabs and the migrated cells located at the reverse side of the chamber were fixed in 4% paraformaldehyde. After being washed with PBS, cells were stained with crystal violet and let stand overnight. Cells that migrated across the filters were counted using a phase-contrast microscope, and the values were averaged.

### Xenograft assay

Four-week old male BALB/c-nu mice were obtained from Charles River Laboratories, Japan, and acclimated for one week. Mice were raised in 22 ± 3 °C with saturated humidity and a standard light/dark cycle. Plenty of food and water was guaranteed.

The same amounts of control and 2FF pre-treated HepG2 cells (4 × 10^6^) were injected subcutaneously into the left and right flanks of each mouse, respectively. Based on the inhibitory effect of 2FF on fucosylation lasted for 7 days at least (Fig. 1C), 100 μl of the DMEM solution containing with 2FF at 100 μM were directly injected into each tumor tissue from three directions after the inoculation for 7 days, and once a week for 3 weeks. All the mice were euthanized at 25 days after the first injection of 2FF. Tumor tissues were isolated, photographed and then weighed. And the same amounts of tumor tissues were homogenized for lectin blot with AAL and Western blot with several indicated antibodies. Measurement of tumor sizes began on the fifth day, and sizes were monitored every 5 days by measuring the tumor length and width with a vernier caliper. Tumor volumes were estimated according to the following formula: volume (mm^3^) = (L × W^2^)/2, where L and W are the length and width of the tumor tissues. All experiments were performed according to protocols approved by the Tohoku Medical and Pharmaceutical University Research Ethics Board.

### Statistical analysis

Statistical analyses were performed using a student’s t-test with GraphPad Prism5 software. Results were presented as the mean ± s.e.m. Statistical significance was accepted at *p* < 0.05. All experiments were repeated at least three times.

## Electronic supplementary material

Supplementary Information
