# Supplementary material for: Inhibition of fucosylation by 2-fluorofucose suppresses human liver cancer HepG2 cell proliferation and migration as well as tumor formation
Source: Sci Rep. 2017 Sep 14;7:11563. doi: 10.1038/s41598-017-11911-9 (PMC5599613; doi:10.1038/s41598-017-11911-9)

Supplementary Information

Inhibition of fucosylation by 2-fluorofucose suppresses human liver cancer HepG2 cell proliferation and migration as well as tumor formation

Ying Zhou[1], Tomohiko Fukuda[1], Qinglei Hang[1], Sicong Hou[1], Tomoya Isaji[1], Akihiko Kameyama[2] and Jianguo Gu[1,]*

[1]Division of Regulatory Glycobiology, Institute of Molecular Biomembrane and Glycobiology, Tohoku Medical and Pharmaceutical University, 4-4-1 Komatsushima, Aoba-ku, Sendai, Miyagi, 981-8558, Japan.

[2]Department of Life Science and Biotechnology, National Institute of Advanced Industrial Science and Technology (AIST), 1-1-1 Umezono, Tsukuba, Ibaraki 305-8568, Japan

*Correspondence and requests for materials should be addressed to J.G. (e-mail: jgu@tohoku-mpu.ac.jp); Tel: +81-22-727-0216; Fax: +81-22-727-0078

Supplementary Figures: Uncropped, full-size scans of western blots illustrated in Fig.1 and Fig.2

Fig 1B

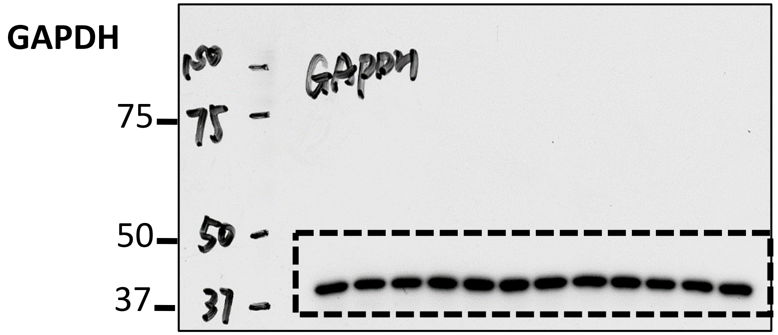

Fig 1C

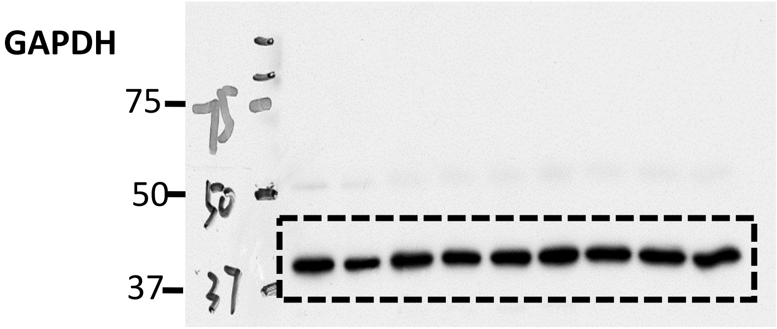

Fig 1D

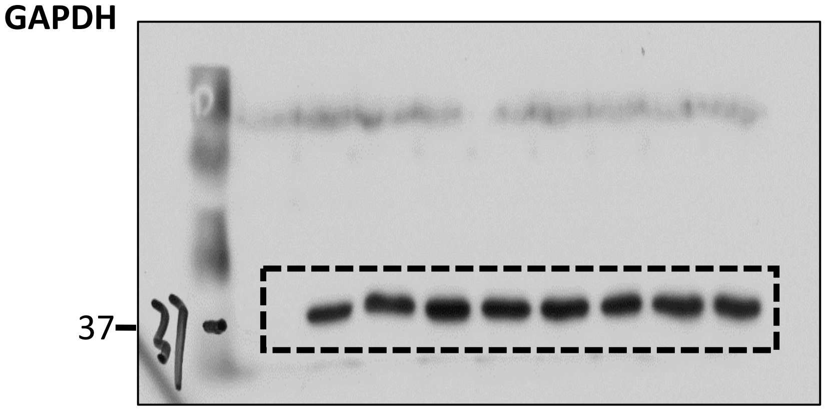

Fig 2B

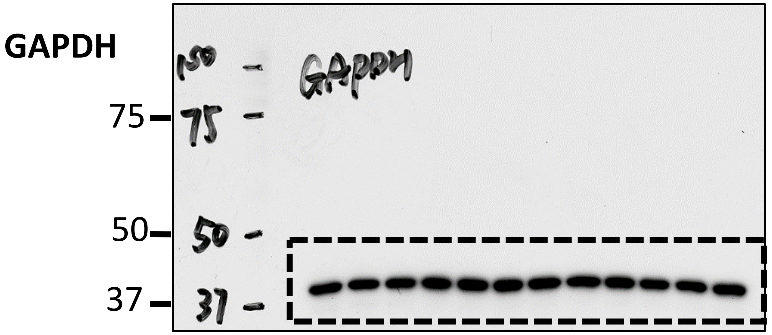

Supplementary Figures: Uncropped, full-size scans of western blots illustrated in Fig.5

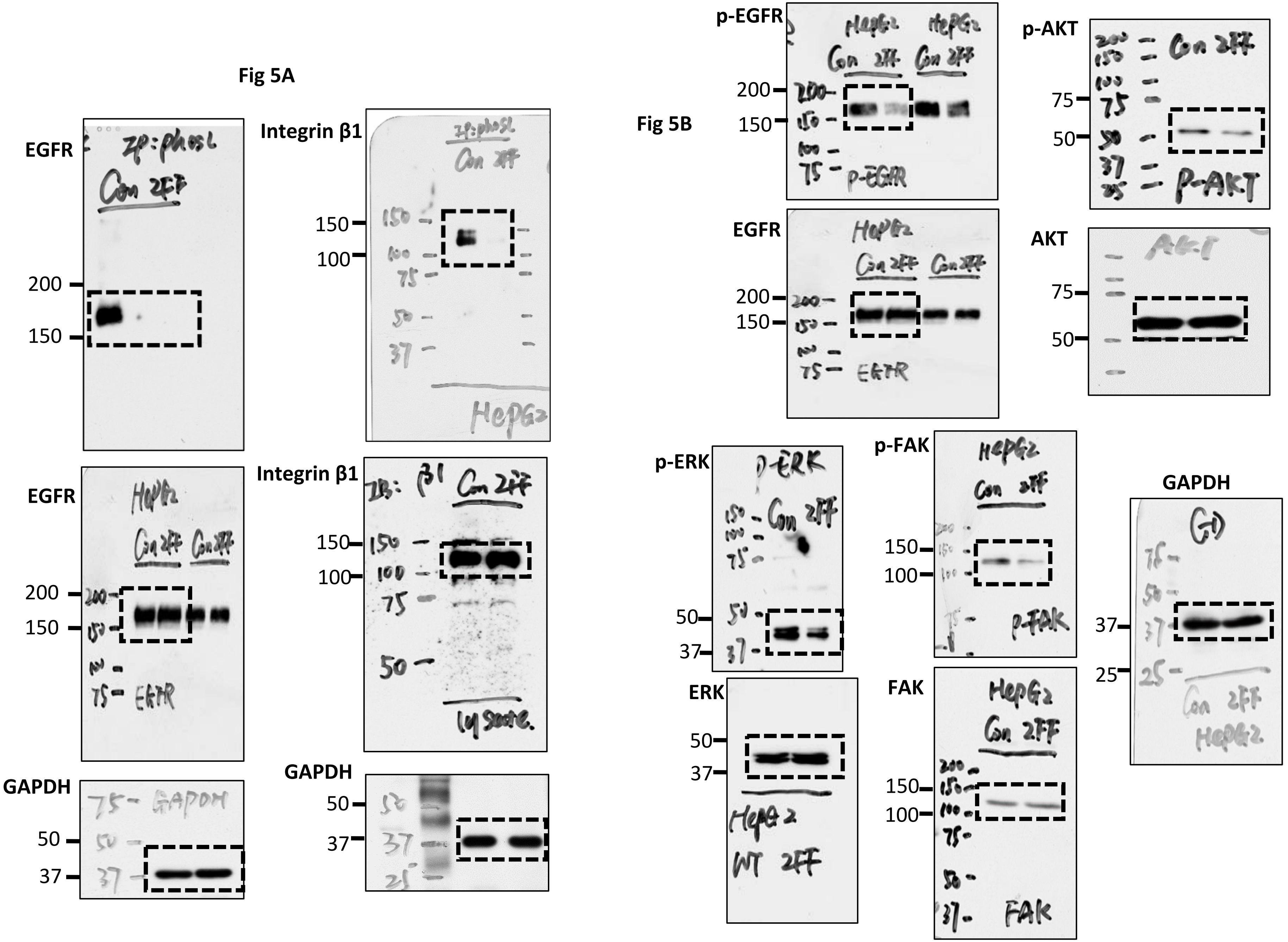

Supplementary Figures: Uncropped, full-size scans of western blots illustrated in Fig.6

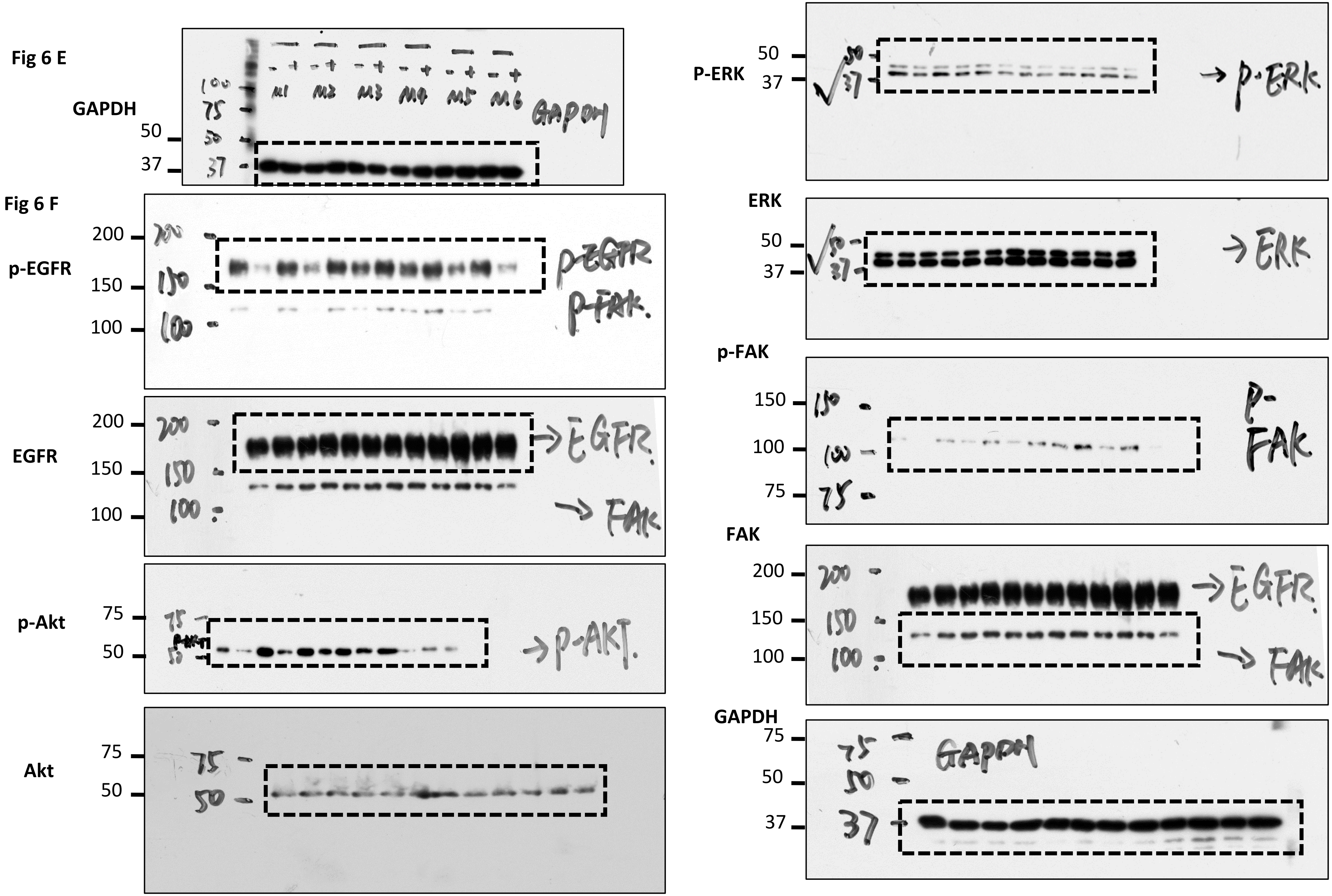

Supplement: Supplementary file 1 — Supplementary Information [file 41598_2017_11911_MOESM1_ESM.pdf]
